# Leucémie à plasmocytes: nouvelles perspectives diagnostiques et thérapeutiques (à propos de cinq cas)

**DOI:** 10.11604/pamj.2025.50.19.45959

**Published:** 2025-01-09

**Authors:** Assya Khermach, Meryem Sabia, Nisma Douzi, Abdelilah Berhili, Mounia Slaoui, Mohammed Bensalah, Rachid Seddik

**Affiliations:** 1 Faculté de Médecine et de Pharmacie, Oujda, Maroc,; 2 Laboratoire d'Hématologie, CHU Mohammed VI, Oujda, Maroc

**Keywords:** Leucémie, plasmocyte, myélome multiple, bortézomib, cas clinique, Leukemia, plasmocyte, multiple myeloma, bortezomib, case report

## Abstract

La leucémie à plasmocytes (LP) est un désordre lymphoprolifératif rare, caractérisée par une prolifération clonale des plasmocytes dans la moelle osseuse et le sang périphérique. En 2021, l'International Myeloma Working Group (IMWG) a redéfini la LP par la présence de 5% ou plus de plasmocytes circulants chez des patients autrement diagnostiqués pour un myélome multiple. Nous rapportons dans ce travail cinq cas de LP colligés au laboratoire d'hématologie du Centre hospitalier universitaire Mohammed VI d'Oujda. L'intérêt de ce travail est d'élucider l'importance de la cytologie hématologique dans l'orientation diagnostique ainsi que l'innovation thérapeutique concernant cette pathologie rare.

## Abstract (en)

Plasma cell leukemia (PCL) is a rare lymphoproliferative disorder characterized by clonal proliferation of plasma cells in the bone marrow and peripheral blood. In 2021, the International Myeloma Working Group (IMWG) redefined LCP as the presence of 5% or more circulating plasma cells in patients otherwise diagnosed with multiple myeloma. In this work, we report five cases of PCL collected in the hematology laboratory of the Mohammed VI University Hospital in Oujda. The interest of this work is to elucidate the importance of hematological cytology in diagnostic guidance as well as therapeutic innovation concerning this rare pathology.

## Introduction

La leucémie à plasmocytes (LP) ou « plasma cell leukemia » est un désordre lymphoprolifératif rare, caractérisé par une prolifération clonale des plasmocytes dans la moelle osseuse et le sang périphérique. Sa fréquence est estimée de 1 à 4%.

Les critères diagnostiques initiaux sont fondés par Kyle en 1974, repris ensuite par l'Organisation mondiale de la Santé (OMS) en 2017, exigeant à la fois une plasmocytose sanguine supérieure à 2G/l ou un taux de plasmocytes supérieur à 20% de la formule leucocytaire [1,2]. Actuellement, la LP est redéfinie par «International Myeloma Working Group» en 2021 par la présence de 5% ou plus de plasmocytes circulants (PC) chez des patients autrement diagnostiqués pour un myélome multiple (MM) [3,4]. La révision de cette définition émane d'une synthèse d'études menées sur des patients myélomateux ou ayant une LP primitive (LPp). En effet, il a été constaté une moyenne de survie similaire pour les patients ayant une plasmocytose sanguine ≥5% avec ceux ayant un taux ≥20%. Cette restriction a permis d'augmenter la sensibilité au diagnostic précoce de la LP, et éventuellement une meilleure prise en charge. On distingue deux formes de LP: la LP primitive ou de novo (60% des cas) survenant d'emblée au diagnostic et la LP secondaire résultant de l'évolution terminale d'un MM (40% des cas). C'est la forme la plus agressive des dyscrasies plasmocytaires. Elle possède des caractères communs avec le MM, mais présente également des particularités cliniques, biologiques et pronostiques. Son pronostic reste sombre malgré de réelles avancées thérapeutiques à l'origine d'un gain de survie.

Nous présentons dans cette étude cinq cas de leucémie à plasmocytes (LP) recueillis au laboratoire d'hématologie du CHU Mohammed VI d'Oujda, Maroc. Parmi ces patients, quatre ayant une LP primitive, tandis qu'un a développé une LP secondaire à l'évolution d'un myélome multiple (MM). À travers ces observations, nous détaillons la physiopathologie de la LP ainsi que ses manifestations cliniques et biologiques, tout en abordant son pronostic. L'objectif de cette étude est de souligner le rôle essentiel de la cytologie hématologique dans le diagnostic de cette pathologie rare et de mettre en évidence son importance dans l'orientation diagnostique.

## Patient et observation

### Observation 1

**Information du patient:** il s'agit d'un patient âgé de 62 ans, maçon de profession, sans tares connues. Dans ses antécédents, on retrouve un tabagisme chronique arrêté il y a 5 mois et exposition au ciment pendant 6 ans.

L'histoire de la maladie débute une semaine avant son admission, avec l'apparition de vomissements intermittents, non liés aux repas, accompagnés de douleurs abdominales péri-ombilicales également intermittentes. Ces symptômes évoluent dans un contexte d'apyrexie et avec conservation de l'état général.

**Résultats cliniques:** à l'examen clinique à l'admission, le patient est conscient, apyrétique, anictérique et présente une sensibilité épigastrique, sans signes d'organomégalie.

**Démarche diagnostique:** un scanner abdominopelvien a révélé un épaississement pariétal irrégulier et asymétrique du duodénum, ainsi qu'un aspect hypertrophié de la tête du pancréas, associé à des adénopathies hilaires, hépatiques et précaves. Il a également mis en évidence des lésions lytiques des os du bassin et de la vertèbre D12. L'hémogramme note une anémie normochrome normocytaire arégénérative à 5,1 G/DL, accompagnée d'une hyperleucocytose à 29 G/L. Le taux des plaquettes est normal. On note également un aspect anormal sur le scattergramme suscitant ainsi la réalisation du frottis sanguin.

**Figure 1:**
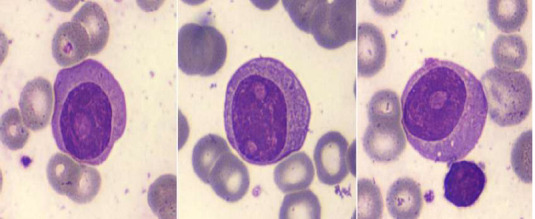
frottis sanguin montrant des plasmocytes circulants, fait majoritairement par des plasmocytes immatures (images du cellavision, DM1200)

L'étude du frottis sanguin avait montré la présence de 20% de plasmocytes circulants faite de plasmocytes matures, de plasmocytes immatures et de quelques plasmoblastes (Figure 1).

L'immunofixation sanguine a montré une bande monoclonale IgG kappa, avec une bande isolée kappa. La recherche d'une bande de type IgD ou IgE faite pour affiner le diagnostic est revenue négative. On a complété ensuite par une immunofixation urinaire qui a objectivé une bande de type chaine légère kappa libre.

**Figure 2:**
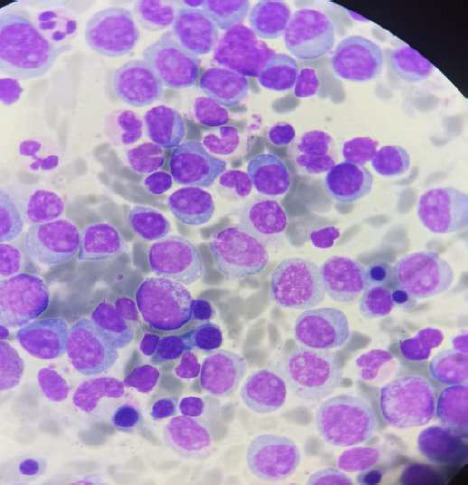
plasmocytose médullaire estimée à 70% avec présence d'un contingent majoritaire de plasmocytes immatures et quelques plasmoblastes. (Fort grossissement G100, MGG)

Le dosage des chaines légères par turbidimétrie a objectivé un rapport K/L augmenté à 11,5. Le myélogramme réalisé ensuite, a décelé l'envahissement par des plasmocytes à 70%, fait de plasmocytes matures à noyaux excentrés, des plasmocytes immatures à chromatine intermédiaire contenant un ou 2 nucléoles et de quelques plasmoblastes (Figure 2). L'immunophénotypage n'a pas pu être réalisé. Quant au bilan biochimique, il a révélé une insuffisance rénale aiguë avec une clairance à 2,8 mL/min, une hyponatrémie à 123 mEq/L, une CRP à 118 mg/L, une franche hyperuricémie à 160 mg/L, une calcémie corrigée à 90 mg/L, une protidémie à 64 G/L et un taux initial de lactate déshydrogénase (LDH) numéré à 448 UI/L.

**Intervention thérapeutique et suivi:** il est pris en charge initialement par le service de néphrologie devant l'insuffisance rénale aiguë où il a bénéficié de 4 séances d'hémodialyse. L'aggravation de sa dyspnée a nécessité la réalisation d'un angioscanner thoracique, qui a objectivé une pneumopathie diffuse interstitielle fibrosante, associée à une pleurésie bilatérale de moyenne abondance. Devant l'aggravation clinique et notamment de l'insuffisance respiratoire aigüe qui est probablement d'origine myélomateux, le patient se fut transféré en réanimation pour prise en charge. Quelques jours après, il a été déclaré décédé.

### Observation 2

**Information du patient:** il s'agit d'un patient âgé de 72 ans, sans antécédents pathologiques particuliers, victime d'une chute de sa hauteur avec réception au niveau de l'épaule gauche.

**Résultats cliniques:** admis au service de traumatologie pour une fracture de la diaphyse humérale.

**Figure 3:**
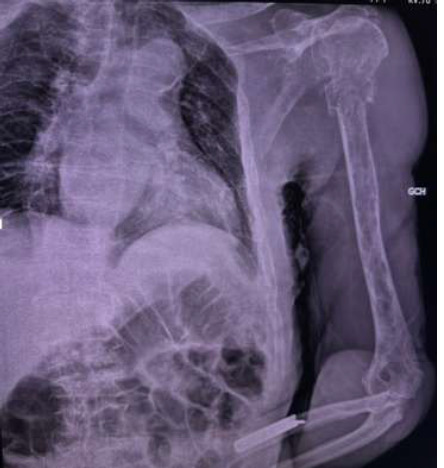
fracture comminutive du col humérale non déplacée sur des images lytiques en pandiaphysite

**Démarche diagnostique:** la radiographie standard de l'épaule gauche a objectivé une fracture comminutive du col huméral non déplacée sur des images lytiques en pandiaphysite (Figure 3). Le patient a bénéficié d'une tomodensitométrie (TDM) thoraco-abdominal qui a montré une déminéralisation osseuse diffuse associée à de multiples lésions osseuses lacunaires intéressant l'ensemble du squelette osseux. Parallèlement, un hémogramme a montré une anémie normochrome normocytaire à 8 g/dl. Un frottis sanguin a été réalisé, montrant une plasmocytose à 8%. Le bilan biochimique a montré une fonction rénale correcte et une calcémie corrigée à 2,7 mmol/l.

**Figure 4:**
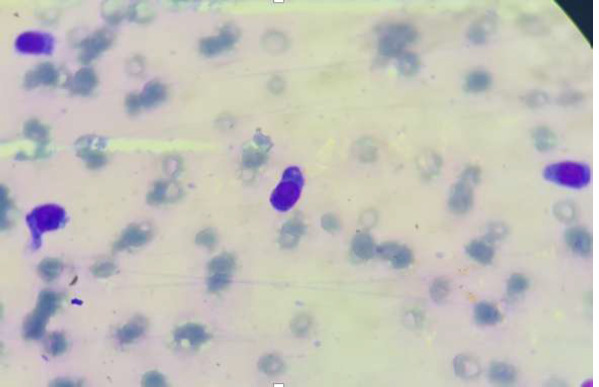
frottis médullaire montrant des plasmocytes matures d'aspect flammé (Fort grossissement G100)

L'électrophorèse des protéines sériques (EPP) note une hypogammaglobulinémie. Une immunofixation sérique complémentaire était négative. Le patient a bénéficié d'un myélogramme qui a objectivé une cellularité faible avec cependant la présence de 35% de plasmocytes d'aspect mature (Figure 4). Vu la faible cellularité médullaire, une biopsie ostéomédullaire a été réalisée. Elle a montré une prolifération plasmocytaire maligne avec une forte expression du CD 138 par l'étude immunohistochimique et une monoclonalité kappa. Le diagnostic de la LP a été retenu.

**Intervention thérapeutique et suivi:** le patient a bénéficié d'une attelle du membre supérieur gauche. Un rendez-vous pour hospitalisation au service de médecine interne lui a été accordé. Cependant, le patient a été perdu de vue.

### Observation 3

**Information du patient:** un patient de 50 ans, sans antécédents notables, est hospitalisé pour des douleurs osseuses diffuses évoluant dans un contexte d'altération de l'état général.

**Résultats cliniques:** l'examen clinique trouve un patient apyrétique, stable sur le plan hémodynamique, sans organomégalie associée.

**Figure 5:**
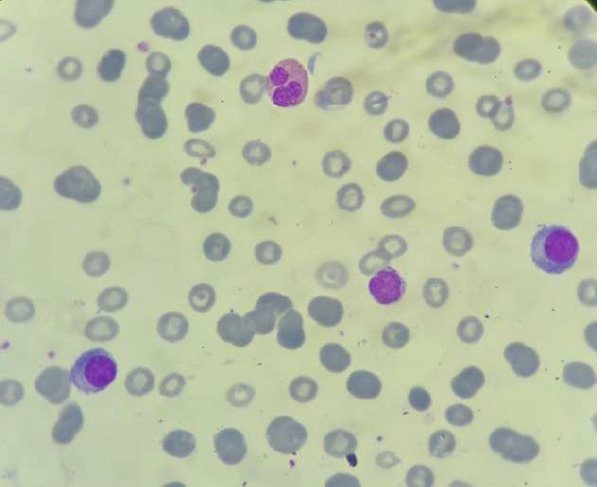
plasmocytose sanguine faite essentiellement de plasmocytes matures avec des hématies en rouleaux

**Démarche diagnostique:** l'hémogramme note une anémie macrocytaire à 3,5 G/DL, une thrombopénie à 137 G/L et une hyperleucocytose à 13 G/l. Devant la bicytopénie, un frottis sanguin a été réalisé montrant une plasmocytose à 27% (Figure 5). Le myélogramme montre une moelle envahie par 90% de plasmocytes d'aspect mature. Parallèlement, un bilan biochimique a été effectué montrant une hypercalcémie corrigée à 3,54 mmol/l, une hyponatrémie à 126 mg/l, une hyperuricémie à 115 mg/l et une insuffisance rénale avec une clairance évaluée à 33 ml/min. L'EPP montre une hypergammaglobulinémie d'allure monoclonale à 29,9 G/L et une hypoalbuminémie à 18 G/L. L'immunofixation sérique a révélé une bande de type IgG kappa. Le diagnostic de la LP a été retenu.

**Intervention thérapeutique et suivi:** le patient a été mis en urgence sous protocole PAD (bortézomide, adriamycine, déxaméthazone) avec une bonne amélioration. Un rendez-vous de contrôle lui a accordé pour évaluer sa réponse au traitement.

### Observation 4

**Information de la patiente:** femme de 54 ans, sans antécédents pathologiques notables, admise pour des douleurs abdominales aiguës accompagnées à des vomissements incoercibles évoluant dans un contexte d'altération de l'état général.

**Résultats cliniques:** l'examen clinique est sans particularité.

**Démarche diagnostique:** sur le plan biologique, l'hémogramme révèle une anémie normochrome normocytaire arégénérative avec un taux de 6,8 G/DL, une thrombopénie à 5,7 G/L, ainsi qu'une hyperleucocytose à 31 G/L. L'analyse du frottis sanguin montre la présence de 37% de plasmocytes, accompagnée de rouleaux d'hématies. Le bilan biochimique met en évidence une insuffisance rénale, avec une clairance de la créatinine évaluée à 11mL/min, une b2-microglobulinémie à 74,41 mg/L, une uricémie à 148 mg/L, ainsi qu'une hypercalcémie corrigée à 3,1 mmol/L et une LDH à 739 UI/L. L'électrophorèse des protéines sériques révèle un double pic au niveau des gammaglobulines, associé à une hypoalbuminémie à 35 G/L. L'immunofixation sérique confirme la présence d'une protéine monoclonale de type IgG kappa. Le myélogramme montre une moelle diluée par le sang périphérique avec présence de 48% de plasmocytes d'aspect mature et un cytoplasme flammé.

**Intervention thérapeutique et suivi:** la patiente a été mise sous protocole PAD. Compte tenu de l'amélioration clinique et de la fonction rénale, la patiente a été déclarée sortie avec un rendez-vous de contrôle programmé.

### Observation 5

**Information de la patiente:** patiente de 64 ans, ayant comme antécédent un diabète type II sous ADO, suivie depuis 2021 pour un MM à chaine légère type lambda.

**Résultats cliniques:** elle s'est présentée aux urgences pour des douleurs osseuses diffuses.

**Figure 6:**
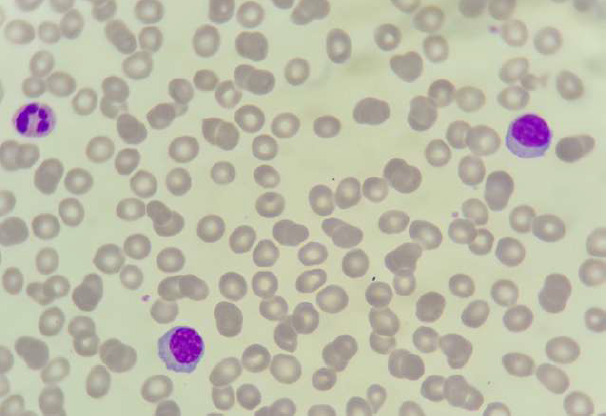
présence de plasmocytes d'aspect mature sur le frottis sanguin

**Démarche diagnostique:** l'hémogramme trouve une anémie normochrome normocytaire à 9,6 G/DL, une thrombopénie à 47 G/L et une leucocytose à 11 G/L. La lecture du frottis sanguin montre une plasmocytose à 15% faite de plasmocyte de petite taille d'aspect mature avec un cytoplasme flammé (Figure 6). Quant au bilan biochimique, il montre une insuffisance rénale avec une clairance à 59 ml/min, une calcémie corrigée à 2,4 mmol/l. L'analyse du frottis médullaire a montré l'envahissement par 93% de plasmocytes.

**Intervention thérapeutique et suivi:** la patiente a reçu la 1^re^ cure de VTP-Bendamustine sans incident. Elle a été déclarée sortante avec un rendez-vous de contrôle pour évaluer l'efficacité thérapeutique.

**Consentement des patients:** le consentement éclairé de chaque patient a été obtenu pour la publication de cet article.

## Discussion

La leucémie à plasmocyte représente la forme la plus agressive des dyscrasies plasmocytaires. Elle est décrite pour la première fois par Foa en 1904 sous le nom de pseudoleucose plasmocytaire, le premier cas rapporté ensuite par Gluzinski Reichenstein en 1906 et c'est qu'en 1946 que Lamy clarifiait ses caractéristiques cliniques et biologiques. C'est une entité rare qui représente entre 2 à 4% des patients atteints de pathologies plasmocytaires. Deux formes à distinguer: la LP primaire ou de novo survenant d'emblée au diagnostic et la forme secondaire compliquant 1 à 4% des myélomes multiples [2].

La LP touche habituellement les hommes avec un ratio H/F de 3/2. L'âge médian au moment du diagnostic est compris entre 49 et 65 ans selon les séries. Nos patients s'insinuent parfaitement dans cet intervalle avec un âge médian de 60 ans.

La cellule myélomateuse (CM) est un plasmocyte des centres germinatifs postérieurs ayant subi une hypermutation somatique, ce qui conduit à la sélection d'une classe spécifique d'immunoglobuline, sécrétée ensuite dans le sérum. Ces cellules adhèrent aux cellules stromales de la moelle osseuse et à la matrice extracellulaire via différentes molécules d'adhésion. Cette adhésion leur permet de résister à l'apoptose, tout en favorisant la libération de cytokines à travers l'activation de diverses cascades de signalisation. Quant à la LP, il a été démontré une expression déficiente du CD56, appelée également *NCAM* (*Neural Cell Adhesion Molecule*), molécule d'adhésion responsable de l'ancrage des CM au stroma médullaire. Une perte d'expression de CD71, CD117 et de l'antigène HLA-DR a été également notée. Ces marqueurs ont été proposés étant discriminant dans les diagnostics difficiles de la LPp par l'*IMWG* [5].

En outre, il a été démontré que l'existence de nombreuses mutations touchant des gènes codant pour les molécules d'adhésion (CD56, I-CAM, N-CAM, LFA…), entrainent ainsi une perte de l'affinité des plasmocytes tumoraux via le stroma médullaire. La t (14;16) par exemple, entraîne l'augmentation de la synthèse d'une métalloprotéase MMP-9 qui est incriminée dans la destruction de la matrice extracellulaire. Ces mécanismes permettent d'expliquer le passage extra médullaire des cellules plasmocytaires [1]. Sur le plan clinique, la LP a une présentation plus agressive que le MM. Les signes cliniques tendent à être une combinaison de ceux trouvés dans une leucémie aiguë et de ceux du MM. Les patients peuvent présenter une infiltration plasmocytaire extra médullaire comme un syndrome tumoral, associant hépatomégalie, splénomégalie, adénopathies, atteinte pulmonaire ou atteinte neuroméningée [2].

La principale symptomatologie décrite est liée à l'insuffisance médullaire et à l'hypercalcémie [1].

Concernant notre série, tous les patients présentaient des douleurs osseuses à l'admission. L'anémie était présente également chez tous nos cas. L'insuffisance rénale, une symptomatologie habituelle dans la LP, est retrouvée dans trois cas. Cependant, aucune localisation extra médullaire n'a été détectée chez nos patients.

Le diagnostic original de la LP est établi en 1974 par Kyle exigent à la fois une plasmocytose sanguine plus de 20% du décompte leucocytaire et un nombre absolu de plasmocytes circulants supérieur à 2×109/l. Cependant, en 2013, le groupe de travail international sur le myélome *IMWG* a signalé que ces critères peuvent être réduits et a demandé que des études soient menées pour réviser les critères diagnostiques de la LP [3].

C'est ainsi que plusieurs études ont été réalisées. En effet, une étude rétrospective espagnole sur 482 patients et publiée en 2017 a objectivé que les patients avec un taux de plasmocytes circulants compris entre 5 et 19% ont une survie similaire à ceux qui ont un taux de plasmocytes ≥20% (c'est-à-dire la LP traditionnellement définie) [6,7]. De même, une étude rétrospective américaine comportant 176 patients et publiée en 2018 a objectivé le même pronostic et la même survie pour des seuils >5% et >20 [7]. En se basant principalement sur ces 2 études, le groupe international de travail sur le myélome multiple (IMWG) a établi en 2021, un critère récent de la LP. En fait, un taux de plasmocytes circulants >5% du décompte leucocytaire suffit pour définir une LP chez les patients autrement diagnostiqués avec un MM symptomatique.

Par conséquent, cette nouvelle définition augmenterait la sensibilité du diagnostic de la LP et à l'adaptation du traitement [4]. Concrètement, les patients ayant une plasmocytose sanguine comprise entre 5 et 20% de la formule leucocytaire sont éligibles pour les traitements et essais cliniques du myélome agressif.

Concernant nos cas de la LPp, c'est l'analyse du frottis sanguin qui a permis de redresser le diagnostic.

Le myélogramme ou la biopsie ostéo médullaire montre un envahissement diffus par des plasmocytes variant entre 50 à 100% [2].

Ces plasmocytes sécrètent une paraprotéine complète ou de chaine légère. Les données de la littérature confirment une prépondérance nette des formes à IgG, suivi par celles à IgA, à chaînes légères, puis par celles de type non sécrétoire, et enfin par celles, exceptionnelles, à IgE [2]. Ce qui concorde avec notre série.

A côté de ses investigations, un bilan biochimique complet doit être effectué. La LDH et la ß2 microglobuline sont significativement plus augmentées dans la LP reflétant ainsi la masse tumorale.

La cytométrie en flux prend toute sa place dans la confirmation de la monoclonalité des plasmocytes mais aussi dans les formes ambiguës où les plasmocytes circulants sont difficiles à identifier.

Les plasmocytes de la LP ont des similitudes immunophénotypiques proches de celui du MM. En effet, on note une expression du CD38 et CD138. Cependant, elle se diffère du MM par l'hyperexpression du CD20, CD117 et une faible expression du CD56 [5,8,9].

L'immunophénotypage n'a pas été réalisé dans notre série, vu la présence de tous les critères définissant la leucémie à plasmocytes.

Sur le plan cytogénétique, la LP est caractérisée par un caryotype plus complexe associant plusieurs anomalies dont les plus fréquemment citées sont la translocation 14q32, la délétion du chromosome 13, la délétion du bras court du chromosome 17. Sans nul doute, ces perturbations cytogénétiques sont impliquées dans la sévérité du tableau Clinique [5].

D'autres anomalies moléculaires ont été détectées: le réarrangement de MYC, les mutations du gène K RAS ou N RAS, anomalies du TP53 et la délétion de PTEN causant l'activation de AKT.

**Figure 7:**
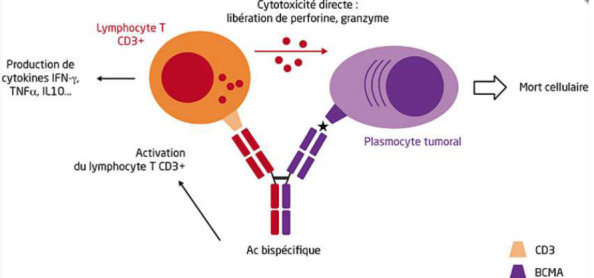
mode d'action des Ac bispécifiques anti-BCMA

Avant l'émergence de nouvelles thérapies, le pronostic était très défavorable, avec une survie inférieure à un an selon la plupart des études. Cependant, grâce à la récente identification de nouveaux marqueurs des dyscrasies plasmocytaires, comme le *BCMA* (*B Cell Maturation Antigen*), il est désormais possible de prévoir qu'au cours des cinq à dix prochaines années, l'introduction de molécules innovantes (telles que les anticorps bispécifiques, les anticorps *ADC* (*Antibody-dependent cell cytotoxicity*), les agents ciblant l'épigénome, ainsi que les nouveaux inhibiteurs du protéasome (IP) et IMiDs) et leur utilisation en combinatoires devraient remplacer l'autogreffe dans le traitement de la LPP (Figure 7) [5].

Le bortézomib, une molécule récemment utilisée, est désormais au cœur du traitement de la leucémie plasmocytaire (LP). Il permet de diminuer la masse tumorale et de réduire les complications associées à cette pathologie [2]. Nos résultats confirment cette efficacité, car les patients ayant reçu du bortézomib ont montré une nette amélioration tant sur le plan clinique que biologique.

Il a également été démontré que la greffe de cellules souches hématopoïétiques (CSH) améliore la survie des patients éligibles [10].

## Conclusion

La leucémie plasmocytaire (LP) est une hémopathie rare et particulièrement agressive. L'avènement de nouvelles thérapies, telles que le bortézomib et les immunomodulateurs, a considérablement amélioré l'efficacité des traitements et la survie des patients. De ce fait, une analyse approfondie du frottis sanguin est essentielle pour surveiller le myélome multiple (MM) et détecter précocement une plasmocytose sanguine dans la LP. Toutefois, malgré les progrès thérapeutiques, la LP demeure une maladie incurable, ce qui souligne l'importance de la cytométrie en flux et de la biologie moléculaire pour le suivi de la maladie résiduelle.
